# Supplementary material for: Effects of Silirum®-Based Vaccination Programs on Map Fecal Shedding and Serological Response in Seven French Dairy Herds
Source: Animals (Basel). 2023 May 8;13(9):1569. doi: 10.3390/ani13091569 (PMC10177616; doi:10.3390/ani13091569)
Supplement: Supplementary file 1 [file animals-13-01569-s001.zip › Corbiere_et_al_Supplementary material.pdf]

Supplementary materials

Effects of Silirum®-Based Vaccination Programs on Map Fecal Shedding and Serological Response in Seven French Dairy Herds

Fabien Corbiere [1,*], Dorra Guellouz [1], Christian Tasca [1], Laurent Foures [2], Emma Dubaux [2] and Gilles Foucras [1]

[1] INRAE, ENVT, UMR 1225 IHAP, 31076 Toulouse, France

[2] GDS Meuse, 55108 Verdun, France

* Correspondence: fabien.corbiere@envt.fr; Tel.: +33561193234

Table S1 : Summary statistics of age at sampling in vaccinated and non-vaccinated cows according to sampling point. For vaccinated cows, time since vaccination (in months) is also reported.

Table S2 : Number of faecal samples according to the estimated Map concentration in the faeces, in each herd, and given the vaccination status of cows

Table S3 : Number of cows according to the estimated Map concentration in the faeces , in each herd, and given their vaccination status. For a given cow, the highest estimated concentration found across its faecal samples was used.

Table S1 : summary statistics (median [first quartile, third quartile]) of age at sampling (in months) in vaccinated and non-vaccinated cows according to sampling point. For vaccinated cows, time since vaccination (in months) is also reported.

	Sampling point			
	1	2	3	4
**Vaccinated cows**				
Number of cows	358	246	34	-
Age	44.9 [42.5; 48.9]	54.5 [50.0; 59.5]	58.8 [53.7; 66.0]	-
Time since vaccination	40.3 [37.0; 43.6]	48.0 [43.6; 55.4]	51.1 [46.5; 58.6]	-
**Non-vaccinated cows**				
Number of cows	265	205	52	3
Age	52.7 [48.7; 57.0]	58.4 [54.7; 62.6]	70.5 [62.1; 76.2]	76.6 [75.9; 78.0]

Table S2 : Number of faecal samples according to the estimated Map concentration in the faeces (in equivalent number of Map per gram of faeces), in each herd, and given the vaccination status of cows

Herd	Vaccination status	Estimated Map concentration (equivalent number of Map.g^{-1})						Overall
		Neg	[5-10^2[	[10^2-10^3[	[10^3-10^4[	[10^4-10^5[	>10^5	
A	Vaccinated	108	6	0	1	0	0	115
	Non vaccinated	117	9	4	0	0	1	131
B	Vaccinated	52	2	0	0	0	0	54
	Non vaccinated	111	9	1	1	0	0	122
C	Vaccinated	64	14	0	0	0	0	78
	Non vaccinated	35	1	0	0	0	0	36
D	Vaccinated	78	5	2	0	0	0	85
	Non vaccinated	76	20	1	1	2	1	101
E	Vaccinated	39	3	0	1	0	0	43
	Non vaccinated	36	4	1	1	0	0	42
F	Vaccinated	102	77	17	11	5	2	214
	Non vaccinated	28	22	8	0	2	1	61
G	Vaccinated	21	23	1	2	1	1	49
	Non vaccinated	17	9	1	1	0	1	29
Overvall	Vaccinated	464	130	20	15	6	3	638
	Non vaccinated	420	74	16	4	4	4	522

Table S3 : Number of cows according to the estimated Map concentration in the faeces (in equivalent number of Map per gram of faeces), in each herd, and given their vaccination status. For a given cow, the highest estimated concentration found across its faecal samples was used.

Herd	Vaccination status	Estimated Map concentration (equivalent number of Map.g^{-1})						Overall
		Neg	[5-10^2[	[10^2-10^3[	[10^3-10^4[	[10^4-10^5[	>10^5	
A	Vaccinated	57	6	0	1	0	0	64
	Non vaccinated	61	9	3	0	0	1	74
B	Vaccinated	26	2	0	0	0	0	28
	Non vaccinated	45	7	1	1	0	0	54
C	Vaccinated	37	14	0	0	0	0	51
	Non vaccinated	19	1	0	0	0	0	20
D	Vaccinated	38	5	2	0	0	0	45
	Non vaccinated	28	17	1	1	2	1	50
E	Vaccinated	18	3	0	1	0	0	22
	Non vaccinated	15	4	1	1	0	0	21
F	Vaccinated	33	52	15	9	5	2	116
	Non vaccinated	9	10	8	0	2	1	146
G	Vaccinated	12	15	1	2	1	1	32
	Non vaccinated	9	5	0	1	0	1	16
Overvall	Vaccinated	221	97	18	13	6	3	358
	Non vaccinated	186	53	14	4	4	4	265
